# Supplementary material for: Hot spot prediction in protein-protein interactions by an ensemble system
Source: BMC Syst Biol. 2018 Dec 31;12(Suppl 9):132. doi: 10.1186/s12918-018-0665-8 (PMC6311905; doi:10.1186/s12918-018-0665-8)
Supplement: Supplementary file 2 — All relevant MATLAB code. A simple Matlab implement of our predictor. (ZIP 19,742 kb) [file 12918_2018_665_MOESM2_ESM.zip › Additional file 2/Read ASA/ASA website/dbMPIKT/4ZGQB.html]

 NetSurfP - Protein Surface Accessibility and Secondary Structure Predictions

  

|  |  |  |
| --- | --- | --- |
|  |  | NetSurfP - Protein Surface Accessibility and Secondary Structure Predictions  Technical University of Denmark |

  


---

```
# For publication of results, please cite:
# A generic method for assignment of reliability scores applied to solvent accessibility predictions.
# Bent Petersen, Thomas Nordahl Petersen, Pernille Andersen, Morten Nielsen and Claus Lundegaard
# BMC Structural Biology 2009, 9:51 doi:10.1186/1472-6807-9-51
#
# Column 1: Class assignment - B for buried or E for Exposed - Threshold: 25% exposure, but not based on RSA
# Column 2: Amino acid
# Column 3: Sequence name
# Column 4: Amino acid number
# Column 5: Relative Surface Accessibility - RSA
# Column 6: Absolute Surface Accessibility
# Column 7: Z-fit score for RSA prediction
# Column 8: Probability for Alpha-Helix
# Column 9: Probability for Beta-strand
# Column 10: Probability for Coil
E D  4ZGQB                  1    0.866 124.848  -0.479   0.003   0.003   0.994
E R  4ZGQB                  2    0.477 109.256  -0.100   0.052   0.084   0.864
B L  4ZGQB                  3    0.169  30.907  -1.588   0.066   0.296   0.638
B V  4ZGQB                  4    0.209  32.169  -0.479   0.069   0.386   0.545
B G  4ZGQB                  5    0.181  14.268  -2.469   0.069   0.386   0.545
E Q  4ZGQB                  6    0.367  65.457  -0.197   0.074   0.484   0.442
B V  4ZGQB                  7    0.119  18.306  -0.689   0.087   0.683   0.230
B V  4ZGQB                  8    0.221  33.983  -0.116   0.022   0.552   0.426
B G  4ZGQB                  9    0.283  22.264  -1.136   0.022   0.359   0.619
E A  4ZGQB                 10    0.396  43.606   0.725   0.020   0.205   0.775
E K  4ZGQB                 11    0.470  96.700  -0.337   0.018   0.088   0.893
E G  4ZGQB                 12    0.616  48.463  -1.490   0.018   0.047   0.935
E H  4ZGQB                 13    0.532  96.680   0.010   0.018   0.047   0.935
B L  4ZGQB                 14    0.244  44.585  -0.216   0.018   0.047   0.935
B P  4ZGQB                 15    0.233  33.006   0.376   0.058   0.017   0.925
E N  4ZGQB                 16    0.540  79.100  -0.707   0.053   0.043   0.903
B I  4ZGQB                 17    0.199  36.741   0.188   0.066   0.296   0.638
B Y  4ZGQB                 18    0.210  44.898   0.536   0.079   0.592   0.329
E T  4ZGQB                 19    0.384  53.316   0.027   0.085   0.775   0.140
E D  4ZGQB                 20    0.472  67.958   0.661   0.072   0.855   0.073
B I  4ZGQB                 21    0.079  14.596   0.382   0.018   0.846   0.136
E E  4ZGQB                 22    0.393  68.570   1.170   0.056   0.913   0.030
B I  4ZGQB                 23    0.074  13.783   0.410   0.056   0.913   0.030
E N  4ZGQB                 24    0.317  46.380   0.691   0.056   0.913   0.030
B Y  4ZGQB                 25    0.109  23.315   0.743   0.072   0.855   0.073
E F  4ZGQB                 26    0.397  79.658   0.898   0.157   0.773   0.069
B L  4ZGQB                 27    0.154  28.234   0.318   0.373   0.401   0.226
B L  4ZGQB                 28    0.080  14.611   0.317   0.649   0.163   0.188
E R  4ZGQB                 29    0.445 101.791   0.652   0.701   0.107   0.192
E R  4ZGQB                 30    0.371  84.867   0.131   0.701   0.107   0.192
B L  4ZGQB                 31    0.112  20.471  -0.102   0.649   0.163   0.188
B L  4ZGQB                 32    0.151  27.703   0.423   0.538   0.173   0.289
E G  4ZGQB                 33    0.348  27.372  -1.046   0.231   0.330   0.439
B V  4ZGQB                 34    0.203  31.201   0.054   0.135   0.317   0.548
E K  4ZGQB                 35    0.489 100.567   0.218   0.069   0.386   0.545
E T  4ZGQB                 36    0.419  58.046  -0.304   0.066   0.296   0.638
E D  4ZGQB                 37    0.607  87.411  -0.700   0.064   0.216   0.721
E G  4ZGQB                 38    0.566  44.544  -0.539   0.052   0.084   0.864
E Q  4ZGQB                 39    0.615 109.910  -0.186   0.053   0.043   0.903
E K  4ZGQB                 40    0.636 130.825   0.401   0.056   0.142   0.802
E Q  4ZGQB                 41    0.588 105.088   0.442   0.069   0.386   0.545
E A  4ZGQB                 42    0.243  26.790  -0.076   0.022   0.552   0.426
E K  4ZGQB                 43    0.507 104.187   1.139   0.022   0.552   0.426
B V  4ZGQB                 44    0.142  21.841   0.346   0.021   0.451   0.528
E R  4ZGQB                 45    0.535 122.401   0.988   0.021   0.279   0.699
E K  4ZGQB                 46    0.496 102.109   1.278   0.066   0.296   0.638
B L  4ZGQB                 47    0.080  14.575  -0.318   0.064   0.216   0.721
E E  4ZGQB                 48    0.509  88.940   0.531   0.056   0.142   0.802
E P  4ZGQB                 49    0.565  80.117   0.914   0.052   0.084   0.864
E N  4ZGQB                 50    0.527  77.080  -0.314   0.018   0.047   0.935
B E  4ZGQB                 51    0.197  34.363   0.001   0.019   0.141   0.840
B V  4ZGQB                 52    0.229  35.228   0.654   0.021   0.756   0.223
B L  4ZGQB                 53    0.047   8.697   0.658   0.011   0.918   0.071
B M  4ZGQB                 54    0.115  23.032   0.568   0.011   0.918   0.071
B V  4ZGQB                 55    0.034   5.210  -0.038   0.011   0.918   0.071
B N  4ZGQB                 56    0.068   9.897  -1.164   0.021   0.756   0.223
B I  4ZGQB                 57    0.059  10.841   0.057   0.022   0.552   0.426
B G  4ZGQB                 58    0.147  11.537  -0.968   0.022   0.359   0.619
B S  4ZGQB                 59    0.233  27.261  -1.045   0.021   0.279   0.699
B T  4ZGQB                 60    0.174  24.134  -1.018   0.064   0.216   0.721
E A  4ZGQB                 61    0.323  35.639  -0.922   0.064   0.216   0.721
B T  4ZGQB                 62    0.301  41.763  -0.937   0.064   0.216   0.721
B G  4ZGQB                 63    0.279  21.965  -1.291   0.066   0.296   0.638
B A  4ZGQB                 64    0.097  10.656  -2.080   0.022   0.552   0.426
E R  4ZGQB                 65    0.277  63.502   0.619   0.011   0.918   0.071
B V  4ZGQB                 66    0.079  12.127   0.031   0.006   0.962   0.032
E V  4ZGQB                 67    0.318  48.815   0.598   0.006   0.962   0.032
E A  4ZGQB                 68    0.383  42.229  -0.083   0.011   0.918   0.071
B V  4ZGQB                 69    0.249  38.241   0.353   0.002   0.816   0.182
E K  4ZGQB                 70    0.457  94.025   0.675   0.004   0.616   0.381
E A  4ZGQB                 71    0.593  65.349  -0.225   0.004   0.138   0.858
E D  4ZGQB                 72    0.603  86.964  -0.854   0.004   0.138   0.858
E M  4ZGQB                 73    0.413  82.561   0.742   0.003   0.718   0.279
B A  4ZGQB                 74    0.062   6.832  -0.253   0.001   0.900   0.099
E R  4ZGQB                 75    0.393  90.066   1.840   0.001   0.959   0.040
B L  4ZGQB                 76    0.049   8.917   1.116   0.001   0.959   0.040
E Q  4ZGQB                 77    0.347  61.956   1.420   0.001   0.959   0.040
B L  4ZGQB                 78    0.063  11.554   0.268   0.001   0.900   0.099
E T  4ZGQB                 79    0.449  62.221   0.689   0.003   0.718   0.279
E S  4ZGQB                 80    0.385  45.087   0.518   0.022   0.359   0.619
B P  4ZGQB                 81    0.198  28.039  -0.387   0.066   0.296   0.638
B A  4ZGQB                 82    0.143  15.803   0.138   0.021   0.451   0.528
B C  4ZGQB                 83    0.122  17.171  -0.189   0.074   0.484   0.442
B T  4ZGQB                 84    0.092  12.802   0.677   0.066   0.296   0.638
E E  4ZGQB                 85    0.509  88.922   0.280   0.056   0.142   0.802
E I  4ZGQB                 86    0.560 103.674   0.925   0.113   0.043   0.844
E N  4ZGQB                 87    0.650  95.219  -1.117   0.058   0.017   0.925
E E  4ZGQB                 88    0.330  57.581  -0.084   0.052   0.084   0.864
E K  4ZGQB                 89    0.395  81.169   1.418   0.087   0.683   0.230
B I  4ZGQB                 90    0.088  16.317   0.740   0.072   0.855   0.073
B A  4ZGQB                 91    0.094  10.337   0.415   0.072   0.855   0.073
B L  4ZGQB                 92    0.101  18.548  -0.149   0.072   0.855   0.073
B S  4ZGQB                 93    0.134  15.693  -0.548   0.085   0.775   0.140
B R  4ZGQB                 94    0.290  66.364   0.041   0.087   0.683   0.230
E R  4ZGQB                 95    0.406  92.928   0.567   0.079   0.592   0.329
B I  4ZGQB                 96    0.163  30.173  -0.253   0.135   0.317   0.548
E E  4ZGQB                 97    0.595 103.999  -0.159   0.052   0.084   0.864
E K  4ZGQB                 98    0.601 123.667   0.252   0.018   0.088   0.893
E H  4ZGQB                 99    0.345  62.719   1.408   0.021   0.279   0.699
B W  4ZGQB                100    0.074  17.701   0.797   0.021   0.756   0.223
B R  4ZGQB                101    0.186  42.571   1.039   0.011   0.918   0.071
B L  4ZGQB                102    0.041   7.599   0.139   0.011   0.918   0.071
B I  4ZGQB                103    0.043   8.029   0.835   0.001   0.900   0.099
B G  4ZGQB                104    0.054   4.218  -1.894   0.002   0.816   0.182
B W  4ZGQB                105    0.129  30.928   0.711   0.003   0.718   0.279
B A  4ZGQB                106    0.054   5.962  -1.227   0.003   0.718   0.279
E T  4ZGQB                107    0.322  44.648   1.062   0.001   0.900   0.099
B I  4ZGQB                108    0.053   9.768   0.457   0.021   0.756   0.223
E K  4ZGQB                109    0.486 100.073   1.326   0.021   0.451   0.528
E K  4ZGQB                110    0.708 145.677   0.461   0.019   0.141   0.840
E G  4ZGQB                111    0.594  46.779  -0.835   0.018   0.047   0.935
E T  4ZGQB                112    0.507  70.376   0.495   0.004   0.138   0.858
E T  4ZGQB                113    0.467  64.787   1.159   0.004   0.420   0.576
B L  4ZGQB                114    0.160  29.259   0.883   0.004   0.514   0.481
E E  4ZGQB                115    0.594 103.737   0.877   0.004   0.420   0.576
E P  4ZGQB                116    0.393  55.781  -0.041   0.004   0.420   0.576
E I  4ZGQB                117    0.349  64.528  -0.572   0.004   0.197   0.799
E A  4ZGQB                118    0.837  92.281  -0.286   0.003   0.003   0.994

Explain the output.  Go back.
```

---
